# Anxiety levels of mothers with newborns in a Neonatal Intensive Care Unit in Turkey

**DOI:** 10.12669/pjms.315.7792

**Published:** 2015

**Authors:** Berrak Mizrak, Ayse Ozge Deniz, Ayfer Acikgoz

**Affiliations:** 1 Berrak Mizrak, RN, MSc. Research Assistant, Eskisehir Osmangazi University, School of Health, Nursing Department, Turkey; 2 Ayse Ozge Deniz, RN, MSc. Nurse, Afyon Zubeyde Hanim Women and Children Hospital, Afyon, Turkey; 3 Dr. Ayfer Acikgoz, PhD. Assistant Professor, Eskisehir Osmangazi University, School of Health, Nursing Department, Turkey

**Keywords:** Anxiety, Healthy newborn, Mother, Sick newborn

## Abstract

**Objective:**

To compare the anxiety levels of mothers with newborns in a Neonatal Intensive Care Unit (NICU) and mothers with healthy newborns in a postpartum care service (PCS).

**Methods:**

This descriptive study was conducted in state and medical school hospitals located in Eskisehir and Afyon, Turkey. The first 200 mothers, including mothers with newborns in a PCS (n=100) and mothers with newborns in a NICU (n=100); participants were followed starting March 1, 2014. Questionnaires to determine the characteristics of mothers and newborns were used as data collection tools, including the State-Trait Anxiety Inventory Scale (STAI TX-1 – STAI TX-2).

**Results:**

Trait anxiety levels were not significantly different between mothers with newborns in the NICU and mothers with newborns in PCS (t=0.588, p=0.557), whereas state anxiety levels were significantly different between the two groups (t=-5.109, p=<0.001). The state anxiety levels of mothers whose infants were in the NICU were determined to be higher compared to those of mothers whose infants were in PCS.

**Conclusion:**

Being a mother of a sick newborn can elevate anxiety and lead to in mothers. During this challenging time, the support of nurses can increase mothers’ abilities to cope with the stress of a sick newborn.

## INTRODUCTION

The Neonatal Intensive Care Unit (NICU) can be a difficult environment for parents because it is noisy, hot and crowded; in addition, advanced medical equipment and complex medical language serve as barriers between parents and their newborns.^1^ Parents, especially mothers, whose newborns are in the NICU can have psychological problems due to having a sick baby, the thought of losing their baby and failure to fulfill traditional parenting roles.^2^-^4^ The lack of a proper environment where mothers can meet their baby’s physiological needs (feeding, drinking, sleeping, etc.), as well as the sickness of their baby, the lack of information regarding treatment and medical operations, the lack of communication with health professionals, not participating in the care of their infants, and the lack of social support can lead to elevated anxiety levels.^5^,^6^

The aim of our study was to compare the anxiety levels of NICU and PCS mothers. We also aimed to determine the factors that can impact anxiety in mothers.

## METHODS

### Sampling

This descriptive study was conducted in state and medical school hospitals located in Eskişehir and Afyon, Turkey. Power analyses were used in order to determine the sample size; as a result of a power analysis based on a previous study by Yurdakul et al., this study determined with 85% power that each group should be composed of 95 mothers.^7^ This study reached 86% power by examining the first 100 mothers in each group (NICU or PCS) who agreed to participate in the study.

### Data Collection Tools

Data collection tools included a descriptive questionnaire form that was created in line with the literature^5^,^7^-^10^ and the State-Trait Anxiety Inventory Scale (STAI TX-1 –STAI TX-2) to determine the anxiety levels of the mothers. The necessary permissions for this study were received from the hospitals. Informed consent was obtained from participants. The descriptive questionnaire form aimed at determining the socio-demographic characteristics of mothers, certain characteristics of the newborns. An additional, the descriptive questionnaire form focused on the opinions of mothers regarding the NICU and the status of their baby in the NICU.

### State-Trait Anxiety Inventory Scale (STAI TX-1 –STAI TX-2)

***1) State Anxiety Inventory Scale:*** This scale determines how an individual feels in a particular place or in a certain condition.
***2) Trait Anxiety Inventory Scale:*** This scale determines how an individual feels regardless of the facts and circumstances.

A Likert-type scale was used that was developed by Spielberger et al. and consisted of 40 items: 20 items measured state anxiety and 20 items measured trait anxiety. Total scores obtained from each scale were evaluated separately. A predetermined and unchanged number was added to this value; this number was 50 for state anxiety and 35 for trait anxiety. The subsequent result was the individual’s anxiety score.^11^ This scale was adapted to Turkish by Öner and Le Compte in 1983.^12^

### Data Collection and Analysis

Written permission and approval were obtained from hospitals and units where this study was conducted. Participants were informed about the aim of the survey, and their verbal informed consent was obtained before the study started. Data collection tools were given to the participants by the researchers during face-to-face interviews. The data obtained from the study were evaluated by the IBM SPSS 21.0 program. In the data analysis, besides descriptive statistics (mean, standard deviation), Student’s t test (in two-group comparisons in which parameters exhibited normal distribution) and the Mann-Whitney U test (in two-group comparisons in which parameters did not follow a normal distribution) were used. Statistical significance was accepted as p<0.05.

## RESULTS

There was no significant difference between the two groups in terms of socio-demographic characteristics (Table-I).

**Table-I:** Socio-demographic Characteristics of Mothers.

Socio-demographic characteristics	Mothers with newborns in a NICU		Mothers with newborns in a PCS		Statistical analysis
	Mean±SD Median (25-75)		Mean±SD Median (25-75)		p
Age	25.69±4.28		25 (23-28)		0.344*
	25.64±5.77		24 (21.25-29)	
	n	%	n	%	x^2^, p
*Educational status*					
Primary school	70	70.0	77	77.0	1.590; 0.452**
High school	23	23.0	16	16.0
University	7	7.0	7	7.0
*Occupational Status*					
Working	13	13.0	8	8.0	0.851; 0.356***
not working	87	87.0	92	92.0
*Income Level Detection*					
Bad	6	6.0	10	10.0	1.238; 0.539**
Moderate	73	73.0	72	72.0
Good	21	21.0	18	18.0
*Supporting Systems*					
Exist	76	76.0	65	65.0	2.909; 0.088**
Not exist	24	24.0	35	35.0
*The Number of Births*					
1	47	47.0	36	36.0	3.727; 0.155**
2	26	26.0	38	38.0
3 or more	27	27.0	26	26.0

* Mann-Whitney U Test,

** Pearson chi-square test,

*** Continuity correction test.

There was a statistically significant difference between the two groups of mothers in state anxiety level scores. The state anxiety levels of mothers whose infants were in the NICU were determined to be higher compared to those of mothers whose infants were in PCS. There was no difference between the groups in trait anxiety levels (Table-II).

**Table-II:** State-Trait Anxiety Levels of Mothers.

	Mothers with newborns in a NICU	Mothers with healthy newborns in a PCS	
	Mean±SD	Mean±SD	Statistical Analysis
State Anxiety Levels	48.20±6.56	43.41±6.61	-5.109▪, <0.001*
Trait Anxiety Levels	40.16±5.48	39.68±5.99	.588, 0.557

▪ Independent samples t-test

* p<0.05

There was no significant relationship between socio-demographic characteristics (age, educational status, occupational status and income level), trait and state anxiety levels of mothers whose infants were in PCS and NICU. A significant relationship was found between presence of supporting systems and state levels of mothers with infants in the NICU. Among mothers whose infants were in the NICU, the ones who reported no support system showed significantly higher state anxiety levels compared to those with support systems (Table-III).

**Table-III:** Distribution of the “State-Trait Anxiety Levels” according to variables of mothers.

		Mothers with newborns in a NICU		Mothers with newborns in a PCS	
		STAI TX-1	STAI TX-2	STAI TX-1	STAI TX-2
Age	r	0.102	-0.110	-0.132	-0.026
	p	0.312	0.275	0.191	0.799

		Mean±SD	Mean ±SD	Mean ±SD	Mean ± SD
*Educational Status*					
Primary School		48.40±6.49	40.03±6.20	43.96±6.91	40.74±5.32
High School		46.37±7.24	38.03±5.46	42.72±6.44	39.73±6.16
University		50.28±5.49	39.57±7.24	40.28±4.46	35.71±1.70
		F=1.009,p= 0.369	F=0.740,p=0.480	x^2KW=.081,p=0.961	x^2KW=2.86,p=0.239
*Occupational Status*					
Working		48.75±6.69	39.04±5.05	42.28±5.39	39.12±4.54
Not working		48.16±6.58	40.32±5.55	43.58±6.87	39.73±6.12
		t=0.242,p=0.809	t=1.460, p=0.155	U=481.50,p=0.388	U=389.50,p=0.784
*Income Level*					
Bad		46.80±7.81	41.37±2.92	43.16±5.11	37.60±6.46
Moderate		49.09±6.73	39.62±5.82	44.02±6.93	40.00±6.31
Good		45.43±4.03	41.67±4.54	41.37±6.03	39.56±4.17
		F=2.580,p=0.081	F=1.294,p=0.279	F=1.288,p= 0.280	x^2KW=0.740,p= 0.691
*Presence of Supporting Systems*					
Yes		45.60±5.68	40.03±5.46	43.16±6.42	39.07±3.79
No		49.60±6.61	39.44±3.84	44.22±7.58	38.01±2.90
		t=3.027,p=0.003*	U=821.00,p=0.462	U=885.00,p=0.827	U=1062.00, p=0.584

* p<0.05

There was no significant relationship between birth weight and gender of the baby with the state-trait anxiety levels of NICU mothers (Table-IV). There was also no association between trait anxiety levels of NICU mothers, and the duration of hospitalisation, the status of getting adequate information about the baby’s situation and the status of participation in the baby’s care (p>0.05); there was a significant link between the state anxiety levels of mothers and these characteristics.

**Table-IV:** Distribution of the “State-Trait Anxiety Levels” according to infant characteristic.

Birth Weight			
	≤2500 gram (n=35)	>2500 gram (n=65)
	Mean ± SD	Mean ± SD
STAI TX-1	48.61±5.70	48.31±7.21	U=1089.00, p=0.725
STAI TX-2	39.89±4.78	40.30±5.85	U=1006.00, p=0.341

Gender of the Baby			
	Female	Male	
	Mean ± SD	Mean ± SD
STAI TX-1	47.97±7.13	48.71±6.42	U=1064.50, p=0.339
STAI TX-2	39.51±6.45	40.59±4.74	t= -.958, p=0.340

Getting information about infant				
	Yes	Partially	No	
	Mean ± SD	Mean ± SD	Mean ± SD	
STAI TX-1	47.64±6.64	48.44±7.00	52.37±5.37	KW=6.367, p=0.041*
STAI TX-2	39.55±3.41	40.21±6.01	40.81±5.39	KW=.246, p=0.884

Participation in the baby’s care				
	Yes	Partially	No
	Mean ±SD	Mean ± SD	Mean ± SD
STAI TX-1	45.47±6.42	46.44±6.14	50.35±7.79	F=3.136, p=0.048*
STAI TX-2	40.42±5.53	40.50±5.77	39.09±5.13	F=.500, p=0.608

* p<0.05

## DISCUSSION

The transition to motherhood is a complex challenge, and having a newborn baby with health problems can be even more complex and difficult.^13^ The state anxiety levels of mothers whose infants were in the NICU were higher compared to PCS mothers, whereas the trait anxiety levels were not different between the two groups (Table-II). There were also other studies whose results were similar to those of our study.^5^,^7^-^9^,^14^

Mothers need psychosocial support during this period in order to ensure the mothers’ emotional balance and decrease negative emotions that can lead to poor health outcomes.^15^ In our study, there was no association between state and trait anxiety levels of PCS, and the presence of support systems; in addition, the state anxiety level was low for mothers with good support systems (Table-II). There were some studies that supported our results.^7^,^9^,^15^,^16^ When we compared the mothers whose infants were in the NICU and PCS, there was no significant relationship between socio-demographic and obstetric characteristics of mothers (age, education status, occupational status income level) and state-trait anxiety levels (Table-III). In line with several results.^7^,^8^ Parents whose babies were admitted to the NICU wanted information, especially from nurses, and interacted with nurses easily.^5^,^17^

In this study, state anxiety levels were higher in mothers whose babies were in the NICU and who did not receive enough information about their infants’ care compared to well-informed mothers (Table-IV). There were different studies that showed getting adequate information about the baby and effective interactions with health professionals led to a decrease in the anxiety levels of mothers.^8^,^10^,^18^ Mothers should be involved in activities such as touching, holding and feeding their babies. In this way, anxiety levels can be decreased via the development of competence and parenting roles.^21^ In our study, the statistically significant mean score of the state anxiety level of mothers who did not participate in the care of their babies (Table-IV). Some other studies have reported similar findings.^19^-^21^

According to our results, there was no significant association between state-trait anxiety levels of mothers and gender of the babies in either group. Erdem (2010), examined mothers whose male infants were in the NICU have high anxiety levels.^8^ In this study, the high anxiety level of mothers with male babies was thought to be an assurance in the future due to the association of gender with religious, cultural and economic understandings and mentalities in Turkey. In our study, the insignificant relationship between anxiety levels of mothers and the gender of the babies was an important result in terms of showing that gender did not contribute to anxiety in the mothers.

## CONCLUSION

Mothers whose babies were hospitalized in the NICU had high levels of anxiety. There were some factors that led to anxiety in mothers such as prolonged periods in the NICU, inability of the mother to care for the baby, and lack of information about the baby’s health. In the literature, studies showed that mothers need to learn about their baby’s status from nurses and interact with nurses freely.
